# Hsp90 *pan* and Isoform-Selective Inhibitors as Sensitizers for Cancer Immunotherapy

**DOI:** 10.3390/ph18071025

**Published:** 2025-07-10

**Authors:** Shiying Jia, Neeraj Maurya, Brian S. J. Blagg, Xin Lu

**Affiliations:** 1 Department of Biological Sciences, Boler-Parseghian Center for Rare Diseases, Harper Cancer Research Institute, University of Notre Dame, Notre Dame, IN 46556, USA; sjia3@nd.edu; 2 Department of Chemistry and Biochemistry, Warren Center for Drug Discovery, University of Notre Dame, Notre Dame, IN 46556, USA; nmaurya@nd.edu; 3 Integrated Biomedical Sciences Graduate Program, University of Notre Dame, Notre Dame, IN 46556, USA; 4 Tumor Microenvironment and Metastasis Program, Indiana University Melvin and Bren Simon Comprehensive Cancer Center, Indianapolis, IN 46202, USA

**Keywords:** Hsp90 inhibitors, Hsp90β, Grp94, isoform-selectivity, cancer immunotherapy, tumor microenvironment, heat shock response, drug development

## Abstract

The 90 kDa heat shock proteins (Hsp90) are molecular chaperones that regulate the stability and maturation of numerous client proteins implicated in the regulation of cancer hallmarks. Despite the potential of *pan*-Hsp90 inhibitors as anticancer therapeutics, their clinical development has been hindered by on-target toxicities, particularly ocular and cardiotoxic effects, as well as the induction of pro-survival, compensatory heat shock responses. Together, these and other complications have prompted the development of isoform-selective Hsp90 inhibitors. In this review, we discuss the molecular bases for Hsp90 function and inhibition and emphasize recent advances in isoform-selective targeting. Importantly, we highlight how Hsp90 inhibition can sensitize tumors to cancer immunotherapy by enhancing antigen presentation, reducing immune checkpoint expression, remodeling the tumor microenvironment, and promoting innate immune activation. Special focus is given to Hsp90β-selective inhibitors, which modulate immunoregulatory pathways without eliciting the deleterious effects observed with pan-inhibition. Preclinical and early clinical data support the integration of Hsp90 inhibitors with immune checkpoint blockade and other immunotherapeutic modalities to overcome resistance mechanisms in immunologically cold tumors. Therefore, the continued development of isoform-selective Hsp90 inhibitors offers a promising avenue to potentiate cancer immunotherapy with improved efficacy.

## 1. Introduction

Heat shock protein-90 (Hsp90) is a ubiquitous, ATP-dependent molecular chaperone that plays a pivotal role in the maintenance of cellular proteostasis. It is highly abundant and comprises 1–2% of total cellular protein, although this can triple (~4–6%) in response to various internal and external stresses [1]. The Hsp90 family includes four highly conserved (>85% identity within the ATP-binding pocket) isoforms, Mitochondrial tumor necrosis receptor-associated protein 1 (TRAP1) localized in mitochondria, glucose-regulated protein 94 (Grp94) localized in the endoplasmic reticulum, Hsp90β (constitutively expressed) and Hsp90α (inducible) in the cytoplasm [2]. All four Hsp90 isoforms are found as dimers and consist of 3-domains [3,4,5]: (i) The N-terminal domain (NTD) contains an ATP-binding pocket wherein hydrolysis occurs [6,7], (ii) the middle domain (MD) which facilitates interaction with client proteins and co-chaperones during the protein folding cycle [8], and (iii) the carboxy-terminal domain (CTD) is responsible for dimerization and the binding of co-chaperones [9]. The C-terminal MEEVD sequence enables the binding of co-chaperones that contain a tetratricopeptide repeat domain [10,11,12]. Furthermore, the C-terminal domain also contains a nucleotide binding site that allosterically regulates the release of ADP from the NTD [9].

**Figure 1:**
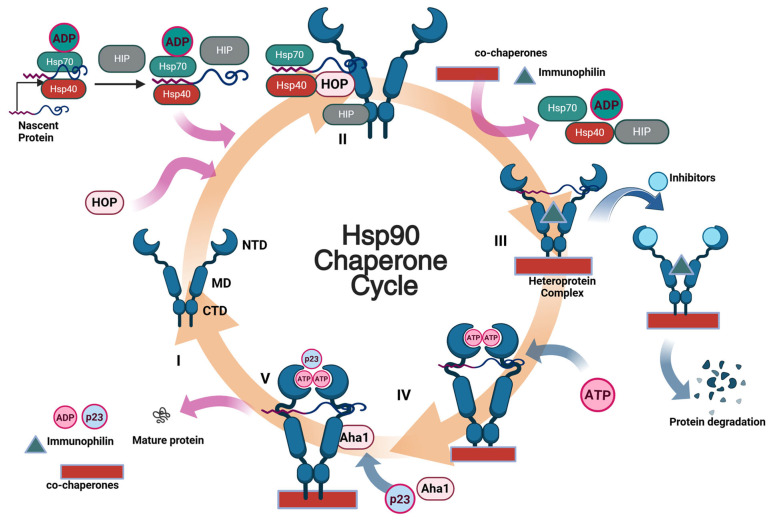
The catalytic chaperone cycle of Hsp90. The cycle begins with the transfer of nascent proteins from the Hsp70-Hsp40 complex (also involving HOP, the Hsp70-Hsp90 organizing protein) to the Hsp90 dimer (**I**,**II**). In Stage (**III**), co-chaperones and immunophilins associate to form a heteroprotein complex with the client. ATP binding induces conformational changes in Hsp90 (**IV**), further stabilized by co-chaperones such as Aha1 and p23, facilitating client protein folding (**V**). Upon ATP hydrolysis, mature proteins are released, and Hsp90 returns to its open conformation (**I**). ATP-competitive Hsp90 inhibitors disrupt this catalytic cycle by preventing proper folding and promoting degradation of client proteins.

Although the process through which Hsp90 facilitates client protein maturation remains to be fully elucidated, it is known to occur via an elaborate protein folding cycle (Figure 1). The cycle begins when Hsp40 and Hsp70 associate with a nascent polypeptide or a misfolded protein to prevent aggregation [13]. Subsequently, the Hsp70-Hsp90 organizing protein (HOP) acts as a carrier to transfer the client protein from Hsp70 to Hsp90 [14]. When co-chaperones and immunophilins bind, Hsp70, Hsp40, and HOP can dissociate to form the active Hsp90 heteroprotein complex (Figure 1 (III)). When ATP binds the Hsp90 NTD, Hsp90 shifts from open to closed conformation (Figure 1 (IV)). Supplemental co-chaperones, such as Aha1 and p23, then bind to the heteroprotein complex. Aha1 stimulates the hydrolysis of ATP, while p23 stabilizes the closed ATP-bound state of Hsp90 (Figure 1 (V)). ATP is then hydrolyzed, which provides the necessary energy to fold the client, disassemble the heteroprotein complex, and regenerate the Hsp90 dimer so that it can re-enter the catalytic cycle (Figure 1 (I)). However, if a small molecule inhibitor binds the NTD instead of ATP, this will cause premature termination of the folding cycle, dissociation of the heteroprotein complex, and the degradation of the client substrate via the ubiquitin-proteasome pathway [15].

**Figure 2:**
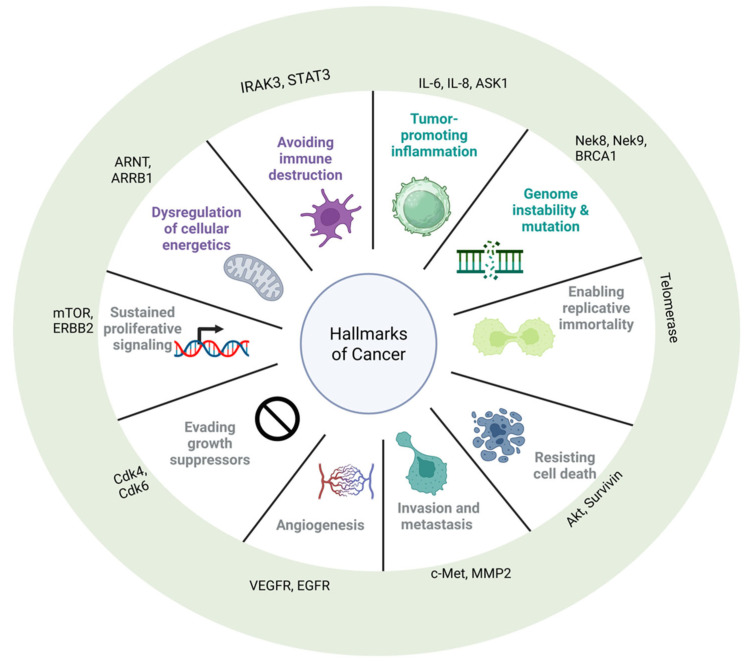
The hallmarks of cancer. As proposed by Hanahan and Weinberg, cancer cells acquire ten hallmark traits that support tumor initiation and progression. Each segment highlights a hallmark alongside client proteins of Heat Shock Protein 90 (Hsp90). For a complete list of the currently identified Hsp90 client proteins see https://www.picard.ch/downloads/Hsp90interactors.pdf (accessed on 16 June 2025).

Maturation, stabilization, and degradation of more than 400 client protein substrates rely upon Hsp90 [16]. Moreover, many of the clients are transcription factors, receptors, kinases, and other oncoproteins that are associated with the hallmarks of cancer (Figure 2; Table 1) [17,18,19,20]. When mutated or overexpressed, these proteins may contribute to cancer progression and drive an oncogenic addiction upon Hsp90. Therefore, the molecular chaperone is an appealing chemotherapeutic target, as its inhibition can simultaneously disrupt multiple oncogenic pathways in a manner that resembles combination therapy. One of the challenges associated with developing anticancer drugs is minimizing off-target effects by selectively targeting cancer cells over normal tissue. Fortunately, data suggest that Hsp90 inhibition can achieve such selectivities, as the heteroprotein complex in tumors exhibits a >200-fold increase in affinity for ATP [21]. Furthermore, unlike kinases and other proteins that utilize ATP, Hsp90 is a member of the GHKL (Gyrase, Hsp90, Histidine Kinase, MutL) family, which contain a unique structural motif called the Bergerat fold forcing ATP to bind in a C-shaped or bent conformation making the development of selective inhibitors feasible [22].

To date, 22 Hsp90 inhibitors have undergone clinical evaluation [23]. Unfortunately, detrimental side effects were observed and have hindered the further development of *pan*-Hsp90 inhibitors. The two most common on-target toxicities, cardiotoxicity and ocular-toxicity, have been attributed to the inhibition of the Hsp90α isoform [24,25]. Therefore, Hsp90 isoform inhibition represents an alternative approach to Hsp90 inhibition without such detrimental attributes. In this review, we discuss the development and evolution of these inhibitors, particularly the development of Hsp90β isoform-selective inhibitors, which represent a new approach toward immunotherapy by sensitizing tumors to immune-based treatments.

## 2. Natural Products as N-Terminal Hsp90 Inhibitors

Efforts to target Hsp90 for the treatment of cancer began in the 1990s. Although conceptually compelling, the idea initially faced considerable skepticism among academic and industrial researchers. This hesitation stemmed primarily from the unprecedented nature of inhibiting a key housekeeping protein; such inhibition might lead to unacceptable systemic toxicities, some of which were validated in subsequent studies [26,27,28,29].

**Figure 3:**
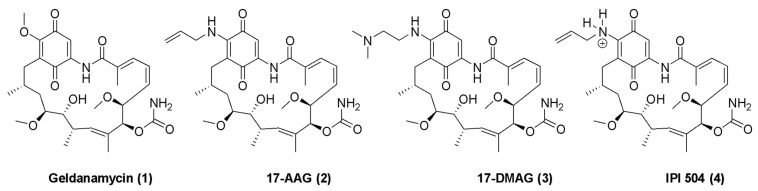
Geldanamycin and its derivatives.

The groundwork for studying Hsp90 was established in the 1970s by the discovery of geldanamycin (GDA), a 1,4-benzoquinone ansamycin antibiotic originally isolated from *Streptomyces hygroscopicus* [30]. GDA was found to reverse the phenotype of v-Src-expressing cells, an effect later attributed to its ability to inhibit v-Src activity indirectly [31,32]. Mechanistically, GDA binds the N-terminal ATP-binding pocket of Hsp90 and induces a conformational change that leads to the destabilization and proteasomal degradation of several oncogenic client proteins, such as Her2, Akt, and v-Src [33]. Despite its ability to inhibit Hsp90, the clinical development of GDA was hindered due to its quinone moiety, which leads to hepatotoxicity in vivo [34,35]. To overcome this limitation, derivatives such as 17-N-allylamino-17-demethoxygeldanamycin (17-AAG) and 17-dimethylamino-ethylamino-17-demethoxygeldanamycin (17-DMAG) were developed (Figure 3). In fact, 17-AAG became the first Hsp90 inhibitor to enter clinical trials, as it demonstrated acceptable tolerability and preliminary signs of therapeutic efficacy in patients. However, challenges such as poor solubility, continued hepatotoxicity, and limited bioavailability eventually led to discontinuation [36].

**Figure 4:**
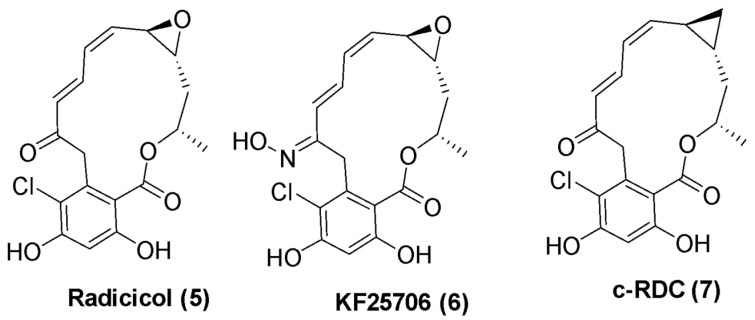
Radicicol and its derivatives.

Radicicol (RDC), a macrocyclic lactone that manifests antifungal activity, was identified as a second natural product that acts via Hsp90 inhibition. It was first isolated in 1953 from fungi *Monocillium nordinii* and *Monosporium bonorden* [37]. RDC was initially suspected to act as a tyrosine kinase inhibitor, but subsequent studies confirmed its inhibitory activity stems from binding the N-terminal ATP-binding site of Hsp90, with a dissociation constant (K_d_) of approximately 17 nM [38,39]. Despite its in vitro potency, RDC exhibited poor in vivo efficacy due to the metabolic instability of reactive moieties such as α, β, γ, δ-unsaturated ketone and the allylic epoxide [40]. To improve upon its pharmacological properties, several RDC derivatives were synthesized, including KF 25706 and c-RDC (Figure 4) [40,41]. The discovery of RDC demonstrated that Hsp90 inhibition is not confined to geldanamycin-like structures. It also validated that the N-terminal ATP-binding pocket is a viable and functionally relevant binding site to manifest Hsp90 inhibition [42]. Importantly, the RDC scaffold provided a structural foundation for subsequent resorcinol-based pyrazole and isoxazole inhibitors that exhibit improved solubility and in vivo potencies. While natural products such as geldanamycin, radicicol, and their derivatives have demonstrated broad anticancer potential, most have failed in clinical trials for reasons discussed below.

## 3. Limitation of Hsp90 *pan*-Inhibition

The development of Hsp90 N-terminal domain inhibitors has faced several challenges in the clinic, resulting in the failure of most inhibitors to date [23]. *pan*-Hsp90 N-terminal inhibitors function by competitively binding to the conserved ATP-binding site shared across all four Hsp90 isoforms. Challenges with previously developed Hsp90 inhibitors include limited efficacy, dose-limiting toxicities, and on-target toxicities, such as ocular and cardiotoxicity. Another problem pan-inhibitors encounter is the induction of the pro-survival heat shock response (HSR), which leads to increased levels of Hsp90. Japan became the first country to approve an Hsp90 inhibitor, pimitespib (TAS-116), to treat advanced gastrointestinal stromal tumors (GIST) [43,44]. A significant difference between pimitespib and earlier Hsp90 inhibitors is that pimitespib selectively targets the cytosolic Hsp90α and Hsp90β isoforms, while Grp94 and Trap1 remain unaffected. Although pimitespib represents a key step toward isoform-selective Hsp90 inhibition, it lacks selectivity between Hsp90α and Hsp90β isoforms [45,46].

## 4. Induction of Heat Shock Response (HSR)

**Figure 5:**
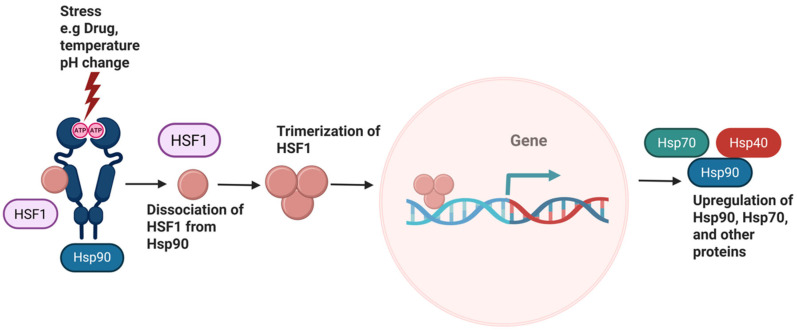
Schematic representation of Heat Shock Response.

Cells activate a protective mechanism known as the heat shock response (HSR) (Figure 5) to maintain cellular proteostasis in response to environmental changes, such as temperature fluctuations, oxidative stress, and pH changes [47]. This pro-survival process is mediated by heat shock factor-1 (HSF-1), one of four transcription factors that can bind the heat shock element (HSE) found within the promoter region for heat shock proteins [48]. In normal cells, HSF-1 remains inactive and associated with Hsp90 [49,50]. When stress arises, either from the environment or by administering an Hsp90 N-terminal inhibitor, HSF-1 dissociates from Hsp90. The unbound HSF-1 trimerizes within the cytosol, becomes phosphorylated, and then translocates into the nucleus, which leads to the transcriptional activation of various heat shock proteins, including Hsp90, Hsp70, and Hsp40 [51,52,53,54,55,56,57]. With the level of Hsp90 increasing following the administration of an inhibitor, even higher levels of Hsp90 inhibitor is needed to combat the Hsp90 activity, resulting in dose-escalating toxicities [58].

## 5. Ocular and Cardiotoxicity

The two significant on-target toxicities observed in clinical trials of Hsp90 inhibitors are ocular and cardiotoxicity, which remain major obstacles to developing Hsp90 inhibitors. The human Ether-a-go-go-Related Gene) (hERG) encodes the alpha subunit of the hERG channel that mediates the rapid repolarization of potassium current (Ikr), which is crucial for the repolarization phase of the cardiac action potential, ensuring the heart beats rhythmically [59]. Hsp90 inhibitors such as geldanamycin impair hERG channel trafficking to the cell surface, leading to acquired long QT syndrome and increasing the risk of cardiac arrhythmias and arrest [28]. In 2012, Peterson and co-workers demonstrated that maturation and trafficking of the hERG channel is solely dependent upon the Hsp90α isoform, which suggests targeting the Hsp90α isoform is detrimental [24].

Another major toxicity observed with *pan*-inhibition is ocular toxicity, observed in both preclinical and clinical settings. In vitro studies demonstrated that *pan*-Hsp90 inhibitors, such as geldanamycin and its analogs, including 17-DMAG, exhibit toxicity toward human retinal pigment epithelial cells [60]. Furthermore, in vivo studies with structurally diverse Hsp90 inhibitors, such as CH5164840 and CH5449302 in beagle dogs, resulted in visual impairments, including loss of pupillary light reflex and abnormal electroretinography (ERG) waveforms [61]. In 2013, Zhou and coworkers determined that prolonged inhibition of Hsp90 in the eye, which results from higher concentrations of 17-DMAG and NVP-AUY922, induces photoreceptor cell death and strong upregulation of Hsp70. Therefore, retinal drug retention is a key factor that contributes to ocular toxicity [25]. This study also showed that the pharmacokinetic profile for Hsp90 inhibitors is important and may indicate the potential for in vivo ocular toxicity. A study published by Aguila et al. in 2014 showed that Hsp90 inhibitors prevent photoreceptor death, but prolonged treatment with inhibitors leads to the degradation of critical client proteins like GRK1 (G protein-coupled Receptor Kinase 1) and PDE6 (Phosphodiesterase 6), which are needed for signaling within the retina [62]. However, a study demonstrated that TAS-116 avoids ocular toxicity because it acts directly on gastrointestinal tumors following administration and has minimal accumulation in the eye, as compared to other inhibitors [46]. Given the intense investigation and recent approval of TAS-116, the development of Hsp90 inhibitors for treating cancer and other diseases, as well as the importance of identifying the isoform-dependent client proteins, are important to identify potential side effects [24].

## 6. Development of Grp94-Selective Inhibitors

**Figure 6:**
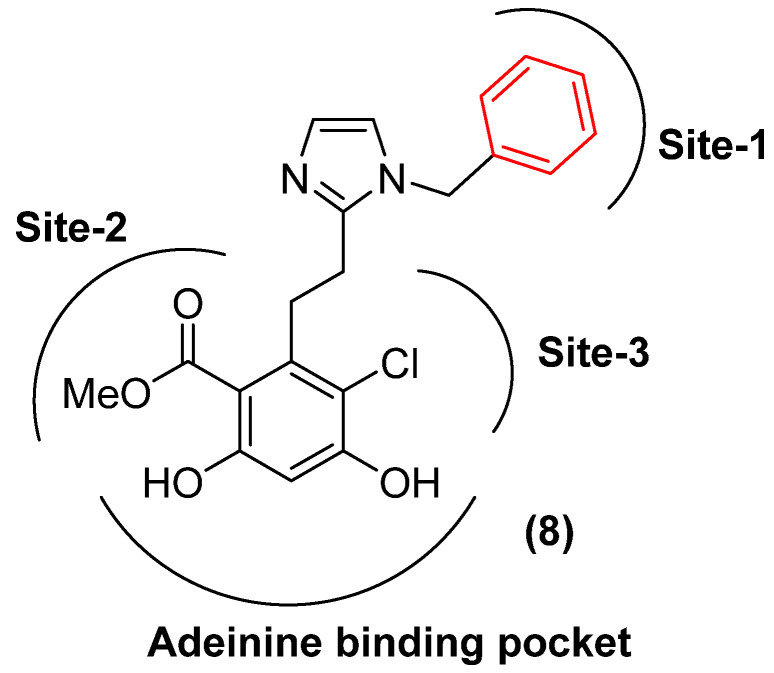
Binding site of Grp94.

**Figure 7:**
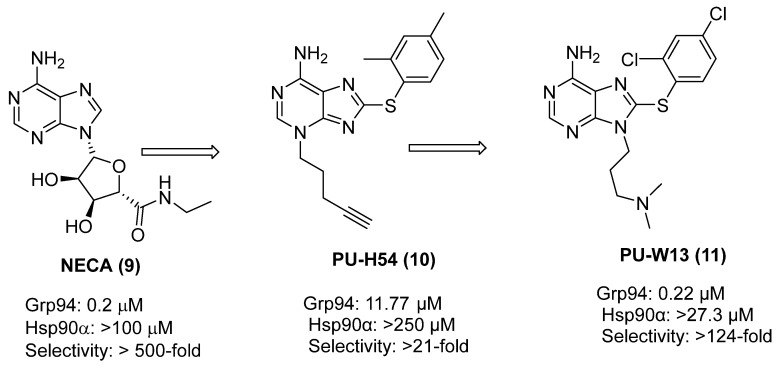
Development of purine-based inhibitors.

Grp94 is an endoplasmic reticulum resident Hsp90 isoform and shares the least identity with other Hsp90 isoforms. Grp94 contains a five-amino-acid insertion (QEDGQ) between residues 182 and 186, resulting in a secondary binding pocket, called site-2 (Figure 6) [63,64,65]. High-throughput screening identified 5′-(N-ethylcarboxamido) adenosine (NECA) as the first Grp94-selective inhibitor that targets the N-terminal ATP-binding domain, which exhibited minimal affinity for Hsp90α [66,67]. However, NECA was not pursued further due to its potent affinity for adenosine receptors, which posed significant off-target liabilities [68]. Several derivatives of NECA were developed to overcome these concerns (Figure 7) [7,69,70].

**Figure 8:**
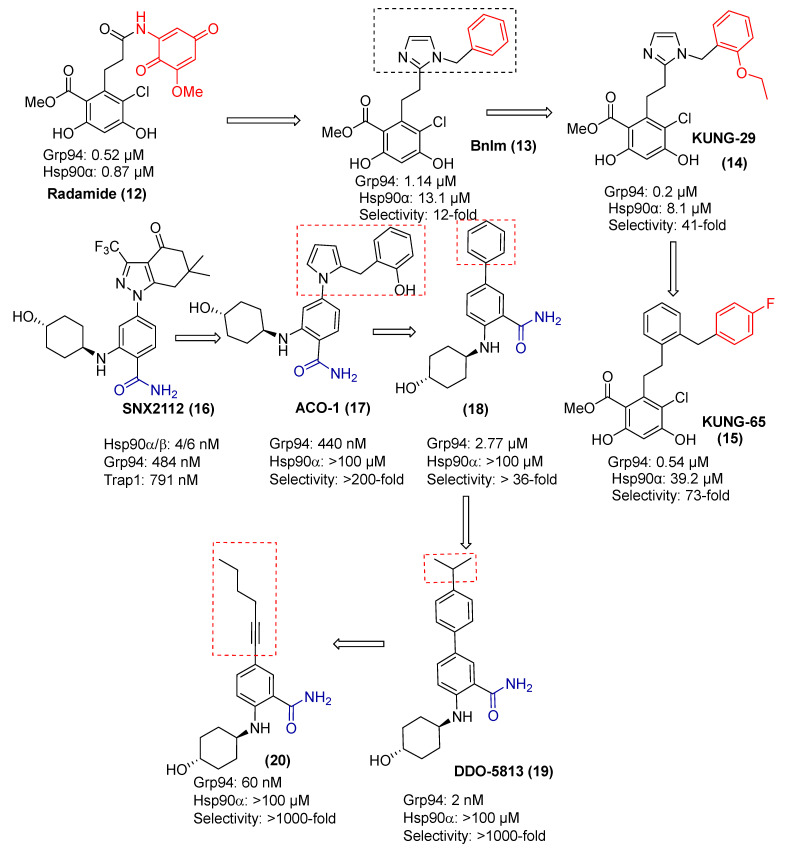
Development of Grp94-isoform selective inhibitors.

Structural studies revealed NECA to bind a distinct sub-pocket within Grp94, referred to as site-3 (Figure 6). Subsequent efforts to exploit this pocket led to the development of radamide (RDA), a resorcinol-based chimera whose quinone moiety engages site-3 through hydrophobic interactions. Replacing the cis-amide of RDA with an imidazole bioisostere and substitution of the quinone with a benzyl group yielded BnIm, which demonstrated enhanced Grp94 selectivity [71]. This improvement was attributed to favorable hydrophobic interactions with Met154, Leu163, Val211, and Trp223. Further SAR studies introduced an ethoxy group at the ortho position of the benzyl ring (KUNG29), which further improved both binding affinity and isoform selectivity. Refinement of KUNG29 by replacing the imidazole with a phenyl ring and appending a para-fluorine substituent led to KUNG65, which exhibited further gains in selectivity (Figure 8) [72,73]. In parallel, a benzamide-based Grp94 inhibitor series derived from SNX-2112 was also developed. Compounds such as ACO-1 and DDO-5813 displayed selectivity greater than 200-fold and 1000-fold for Grp94, respectively, with significant contributions from modifications of the aryl and linker regions.

## 7. Development of Cytosolic Benzamide-Scaffold Hsp90 Inhibitors

The cytosolic isoforms of Hsp90, Hsp90α, and Hsp90β share approximately 95% sequence identity within their N-terminal ATP-binding domains, which has made discrimination by small molecule inhibitors a significant challenge [74]. In the early 2000s, efforts to overcome the limitations associated with natural product-derived Hsp90 inhibitors led to the development of synthetic small molecules with enhanced pharmacological properties. Among these, SNX-2112, developed by Serenex Inc., featured a benzamide scaffold and demonstrated improved pharmacokinetics [75]. SNX-2112 and its prodrug SNX-5422 have been shown to promote the degradation of several oncogenic client proteins, including HER2, Akt, and Raf-1 [76]. However, pharmacokinetic studies revealed a substantial accumulation of SNX-2112 in the liver and kidneys, raising concerns about off-target toxicity [77]. Moreover, SNX-2112 exhibited limited isoform selectivity between Hsp90α and Hsp90β, resulting in adverse effects and hindering its clinical development [78].

**Figure 9:**
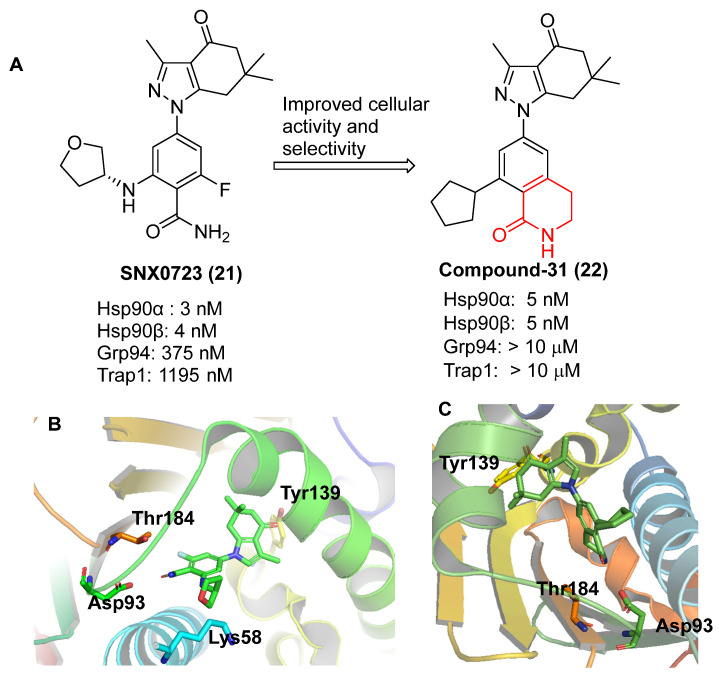
(**A**) Chemical structure of SNX0723 and Compound **31**. (**B**) Binding mode of SNX0723 to Hsp90α NTD (PDB code: 4NH8) (**C**) Binding of compound **31** to Hsp90α NTD (PDB code: 4O0B).

Between 2008 and 2014, Serenex Inc. and Vertex Pharmaceuticals continued to develop synthetic Hsp90 inhibitors with improved isoform selectivity. Among them, SNX0723 has shown over 100-fold selectivity for both cytosolic Hsp90α/β, as compared to the endoplasmic reticulum and mitochondrial paralogs, Grp94 and TRAP1 [79]. Further structural refinement led to the discovery of Compound **31**, a benzyl-lactam–based scaffold with >1000-fold selectivity for Hsp90α/β over the other isoforms (Figure 9). Structural analysis revealed that selectively binding to the cytosolic isoforms is driven by these ligands’ ability to engage the 104–111 amino acid region within the N-terminal ATP-binding domain, which adopts an α-helical conformation unique to Hsp90α/β. In contrast, Grp94 and TRAP1 cannot accommodate this helical structure, resulting in weaker or no binding of inhibitors [79]. This confirmation is crucial for achieving selectivity for the cytoplasmic Hsp90 isoforms [80]. Notably, Compound **31** can penetrate the blood–brain barrier, and is currently under investigation for potential applications in neurodegeneration such as Huntington’s disease [79]. Another compound, TAS-116 (pimitespib), incorporates a benzamide group and also exhibits selectivity for both cytosolic Hsp90 isoforms [81]. Despite these advances, the development of isoform-selective inhibitors that discriminate between Hsp90α and Hsp90β remained a significant challenge, as the two isoforms share extensive sequence identity, differing only at Ala52 and Leu91 in Hsp90β versus Ser52 and Ile91 in Hsp90α within the N-terminal ATP-binding pocket.

## 8. Development of Hsp90β-Selective Inhibitors

While early compounds demonstrated selectivity for the cytosolic Hsp90 isoforms, the inability to discriminate between Hsp90α and Hsp90β remained a significant limitation. Inhibition of Hsp90α has been associated with on-target toxicities, including ocular and cardiac side effects, thus underscoring the need for isoform-specific strategies to avoid these detriments.

**Figure 10:**
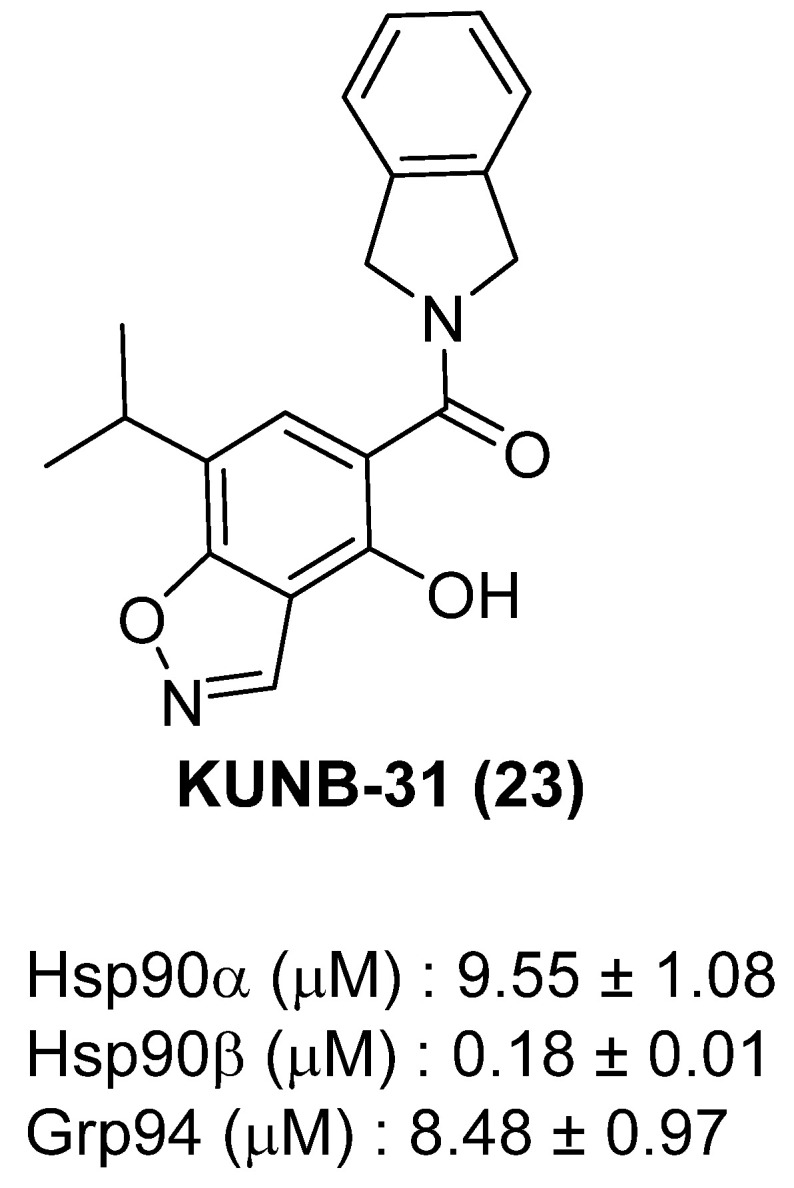
Chemical structure of KUNB31.

By the mid-2010s, evidence had accumulated that Hsp90 isoforms have both distinct and overlapping cellular functions. For example, c-Raf and the hERG channel are clients that depend solely upon Hsp90α, while c-IAP1, Cdk6, and Cxcr4 depend upon Hsp90β for maturation [24]. Functionally, Hsp90α is stress-inducible and plays a role in extracellular signaling, wound healing, and metastatic progression, while Hsp90β is constitutively expressed and essential for embryonic development, cellular metabolism, and the stabilization of key immunoregulatory proteins, including PD-L1, STAT3, and IDO1 [82,83,84]. Such observations provided a foundation for the development of isoform-selective inhibitors, particularly for Hsp90β, to enable more targeted tumor suppression with fewer side effects. In 2018, Khandelwal and coworkers reported the first Hsp90 N-terminal isoform selective inhibitor, KUNB31 [85]. This was achieved by a series of structure-based studies and systematic modification of the resorcinol ring to give an isoxazole ring containing inhibitor, KUNB31, which binds selectively to Hsp90 N-terminal binding site with Kd~180 nM and ~50-fold selectivity over Hsp90α (Figure 10). KUNB31 induced degradation of several Hsp90β-dependent client proteins, such as c-IAP1, Cdk6, and Cxcr4, while avoiding induction of Hsp90 levels. This was the first demonstration that cytosolic isoforms can be selectively targeted, despite having high identity, without inducing a Hsp90 levels or inhibiting hERG maturation [85].

**Figure 11:**
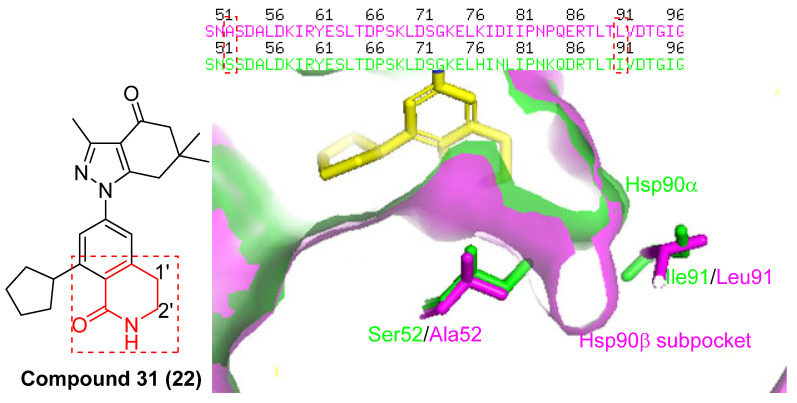
Compound **31** docked into the N-terminal ATP-binding pocket of Hsp90β (Magenta surface) and Hsp90α (green surface) PDB code: 1UYM and 4o0b.

**Figure 12:**
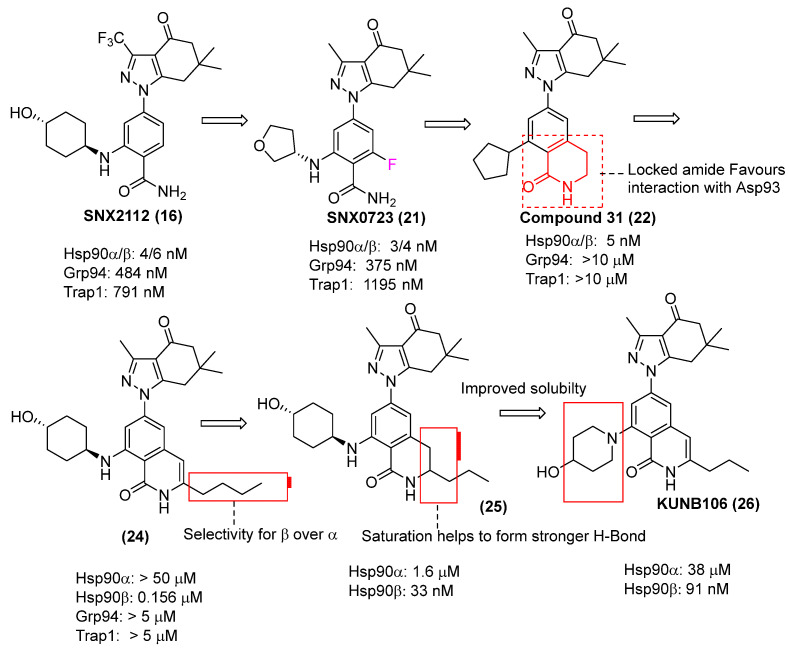
Development of Hsp90β-selective inhibitors and its optimization.

A more recent breakthrough came from Mishra and colleagues, who developed highly potent isoquinolinone-containing Hsp90β-selective inhibitors in 2021 [74]. These efforts began with an overlay of two co-crystal structures: one of Hsp90β (PDB: 1UYM) and one of Compound **31** bound to Hsp90α (PDB: 4O0B) (Figure 11), from which several important observations were made. First, it was noted that, going from isoleucine in Hsp90α to leucine in Hsp90β, a greater flexibility occurred, resulting in a small, hydrophobic sub-pocket found exclusively within Hsp90β. Therefore, it was proposed that occupation of this pocket via substitution about Compound 31’s lactam ring could drive out conserved water molecules at the bottom of the ATP-binding pocket and provide Hsp90β selectivity. This hypothesis was ultimately validated, as several compounds with nanomolar affinities towards Hsp90β were reported (Figure 12) [74].

The second crucial observation from this overlay was that the cyclopentyl ring of Compound **31** occupies a solvent-exposed region of the binding pocket. Therefore, appendages at this position can improve affinity and selectivity via interactions with water molecules and/or gateway residues. Furthermore, bulkier appendages could not only be tolerated at this region due to a lower chance of steric clashing, but they might also serve as a “plug” to block water molecules from accessing the pocket and further strengthen protein-ligand interactions. The first moieties explored at this location of the scaffold were anilines, but they facilitated inter-/intramolecular hydrogen bonding interactions that increase the planarity of these inhibitors. This, in combination with their numerous aromatic rings, contributed to poor aqueous solubility due to persistent π–π stacking interactions, which contribute to a strong crystal lattice energy [86].

**Figure 13:**
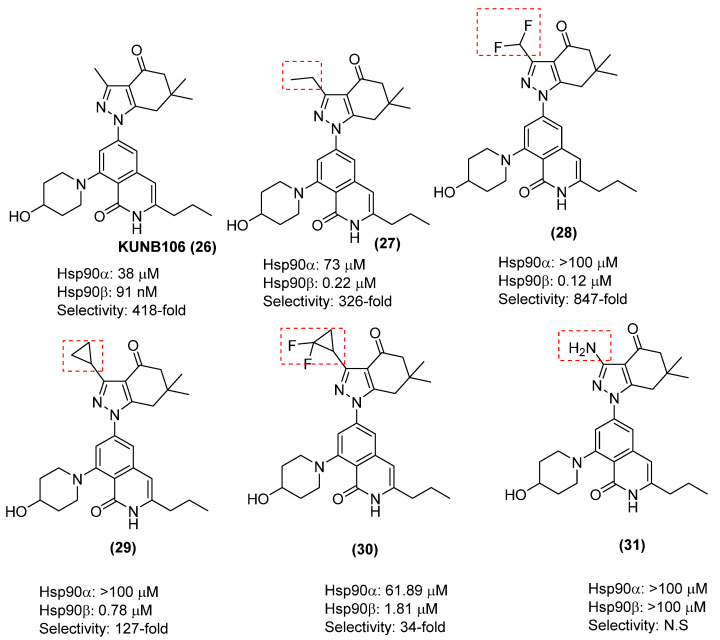
An SAR study on the pyrazole methyl of KUNB106 (N.S = Not significant).

Consequently, a series of SAR studies were performed along several regions of the KUNB106 (**26**) scaffold to tune the selectivity, affinity, and solubility. In one study, modifications to five regions of the indazolone ring system (the pyrazole methyl, the pyrazole core, the carbonyl, the a-keto position, and the gem-dimethyl group) were prepared and evaluated (**27**–**39**). SAR study on the pyrazole core was performed (Figure 13), starting with the replacement of methyl with an ethyl substituent (**27**), which resulted in a decrease in both selectivity and affinity. Compound **28**, with a difluromethyl group, led to an increase in selectivity of >817-fold. Compound **29**, which bears a cyclopropyl group, manifested an apparent binding affinity for Hsp90β = 0.78 μM, but reduced selectivity to 127-fold. When the fluoro group (**30**) was introduced on to the cyclopropyl ring, it resulted in both a loss in affinity and selectivity. An interesting observation was that when the aniline was introduced (**31**), it did not manifest measurable affinity for either isoform. This SAR study demonstrated that even subtle modifications on the pyrazole ring can significantly impact affinity and selectivity [86].

**Figure 14:**
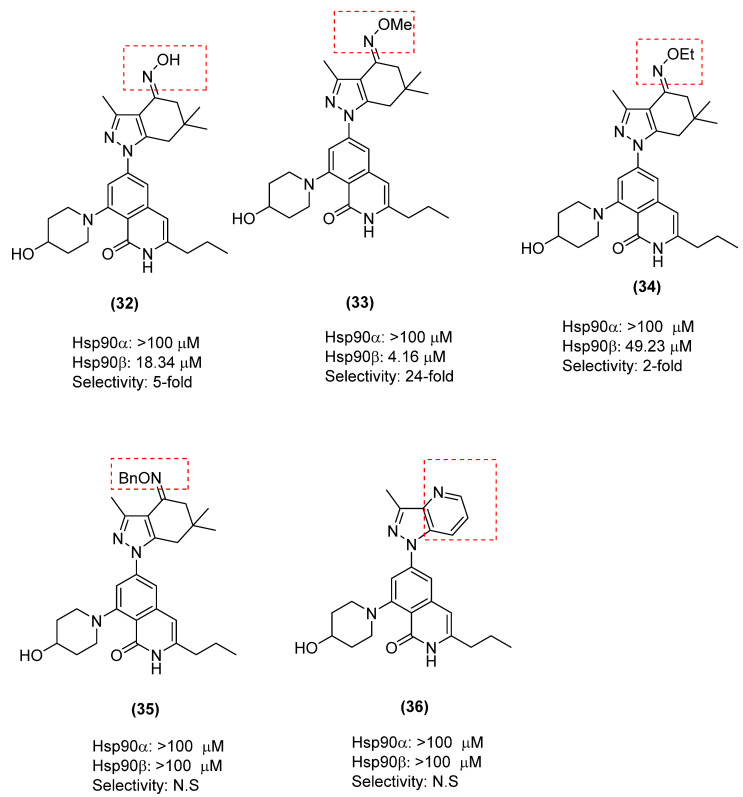
An SAR study on ketone moiety of KUNB106, (N.S = Not significant).

When SAR studies were performed on the ketone moiety, interesting results were obtained, which were consistent with an earlier study that had suggested that this carbonyl maintains a key hydrogen bond interaction with Tyr139. The oximes were installed to determine whether space is accessible in that region of the pocket. The pyridine is an H-bond acceptor similar to the carbonyl oxygen; therefore, it was prepared to determine whether the H-bond acceptor needed to be exocyclic. It was hoped that the pyridine could improve affinity by binding to Tyr and effectively cause the protein to more tightly “clamp down” on the inhibitor. Replacement of this group with an oxime in (**32**–**36**) resulted in a significant loss in affinity for Hsp90β and selectivity for Hsp90β over Hsp90α (Figure 14) [86].

**Figure 15:**
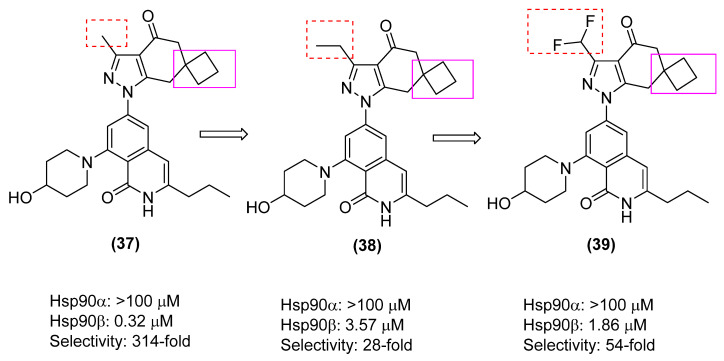
An SAR study on the dimethyl group of KUNB106.

An SAR study determined the importance of the dimethyl group in KUNB106 (Figure 15); the dimethyl group was replaced with a spirocyclic cyclobutane, as shown in (**37**–**39**), methyl was replaced with an ethyl, and difluoromethyl was also evaluated to see if this moiety affects affinity and selectivity. Across all these analogs, none showed improved activity nor selectivity over the parent compound, KUNB106. Ongoing efforts will continue to develop better Hsp90β inhibitors [86].

## 9. A Brief Recount of Cancer Immunotherapy

**Figure 16:**
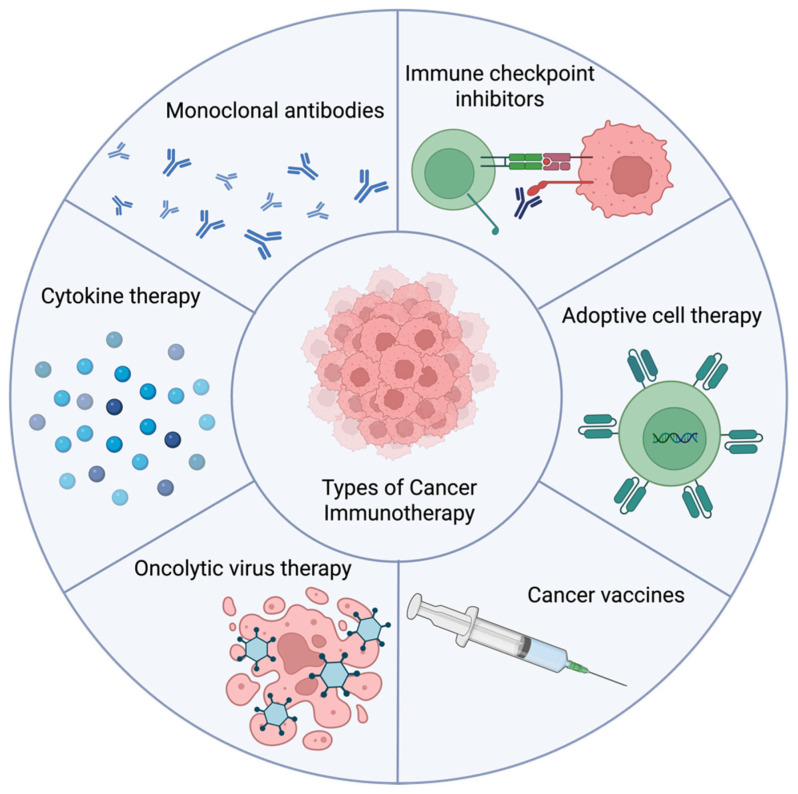
Major types of cancer immunotherapy. Cancer immunotherapy includes a variety of strategies, such as immune checkpoint inhibitors, adoptive cell therapy, cancer vaccines, oncolytic viruses, cytokine therapies, and monoclonal antibodies. These approaches engage the immune system in different ways to help recognize and eliminate tumor cells.

The idea of harnessing the immune system to combat cancer, now known as cancer immunotherapy, can be traced back to the late 19th century. German physicians Wilhelm Busch and Friedrich Fehleisen were among the first to document a potential link between immune activation and tumor regression, observing that erysipelas infections, typically caused by *Streptococcus pyogenes*, sometimes led to tumor shrinkage [87]. Building on these observations, William Coley, often regarded as the “Father of Cancer Immunotherapy”, developed bacterial extract mixtures—known as Coley’s toxins—to provoke immune responses in patients with sarcoma [88]. Although his approach was controversial at the time and eventually fell out of favor, the core concept of stimulating the immune system to target cancer persisted. Interest in immunotherapy re-emerged in the 20th century, driven by significant advances in immunology and a growing understanding of the complex relationship between tumors and the immune system [89]. A landmark moment came in 2018 when James P. Allison and Tasuku Honjo were awarded the Nobel Prize in Physiology and Medicine for their discovery of cancer therapy through inhibition of negative immune regulation, underscoring the clinical potential of immune modulation in oncology [90,91,92,93,94,95,96]. Tremendous progress has been made in this field over the past decades; cancer immunotherapy has evolved into a cornerstone of modern oncology, encompassing a broad array of strategies such as cytokine therapies, monoclonal antibodies, immune checkpoint inhibitors (ICIs), adoptive cell transfer (ACT) approaches including chimeric antigen receptor T-cell (CAR-T) therapy, cancer vaccines, and oncolytic virus therapies (Figure 16), many of which have achieved remarkable clinical success across various malignancies [97,98,99,100,101,102].

However, despite these remarkable advances, the realization of the full potential of cancer immunotherapy still faces formidable challenges. Current biomarkers such as PD-L1 expression, microsatellite instability (MSI), and tumor mutational burden (TMB) provide some guidance for patient selection but remain insufficiently reliable for accurately identifying individuals most likely to benefit from immunotherapy [103,104,105,106,107]. Immune-related adverse events (irAEs) impose additional restrictions on wider clinical applications [108]. Another significant limitation is the heterogeneity of patient response; only a minority of patients experience durable clinical benefits [109,110,111]. A large subgroup of patients fail to respond entirely or relapse after initial treatment success, underscoring resistance as a central unresolved issue in cancer immunotherapy. Such resistance can be categorized into primary (intrinsic) resistance, where tumors do not respond initially, and acquired resistance, where patients who respond ultimately progress. These forms of resistance originate from the complicated interactions between tumor-intrinsic factors and tumor-extrinsic factors, such as an immunosuppressive tumor microenvironment (TME).

Tumor-intrinsic resistance arises from a spectrum of genetic, epigenetic, and metabolic alterations that collectively enable immune evasion. Key mechanisms include the loss or downregulation of neoantigens and tumor-associated antigens [112,113,114,115,116,117,118], defects in antigen processing and presentation machinery [119,120,121,122], and constitutive expression of immune checkpoint ligands such as PD-L1. Aberrant activation of signaling pathways, including WNT/β-catenin [123], PI3K-AKT [124], and MAPK [125], promotes immune exclusion partially by impairing dendritic cell recruitment and suppressing local T cell priming. Mutations that disrupt interferon-γ signaling pathway reduce tumor sensitivity to the cytotoxic cytokine [126]. In parallel, epigenetic silencing of pro-apoptotic genes, antigen presentation machinery, and tumor antigens facilitates immune escape [127,128,129,130,131]. Metabolic reprogramming within tumor cells, characterized by enhanced glycolysis, tryptophan catabolism via indoleamine 2,3-dioxygenase (IDO), adenosine accumulation, and hypoxia, contributes to the formation of an immunosuppressive TME, impairing T and NK cell function [132]. In addition, the TME is often enriched with immunosuppressive cell populations, including regulatory T cells (Tregs), myeloid-derived suppressor cells (MDSCs), tumor-associated macrophages (TAMs), and cancer-associated fibroblasts (CAFs), all of which impede effective antitumor immunity. Beyond the tumor and its microenvironment, host-related variables, such as gut microbiota composition, systemic inflammatory states, and host-specific genetic and/or epigenetic landscapes, have also been shown to modulate the response to immunotherapy [133,134,135].

New approaches to improve the clinical efficacy of cancer immunotherapies are urgently required. Of close relevance to this review, an increasing body of data suggest that Hsp90 inhibitors can sensitize tumors to immunotherapy through a variety of mechanisms.

## 10. Mechanisms of How Hsp90 Inhibitors Enhance Cancer Immunotherapy

**Figure 17:**
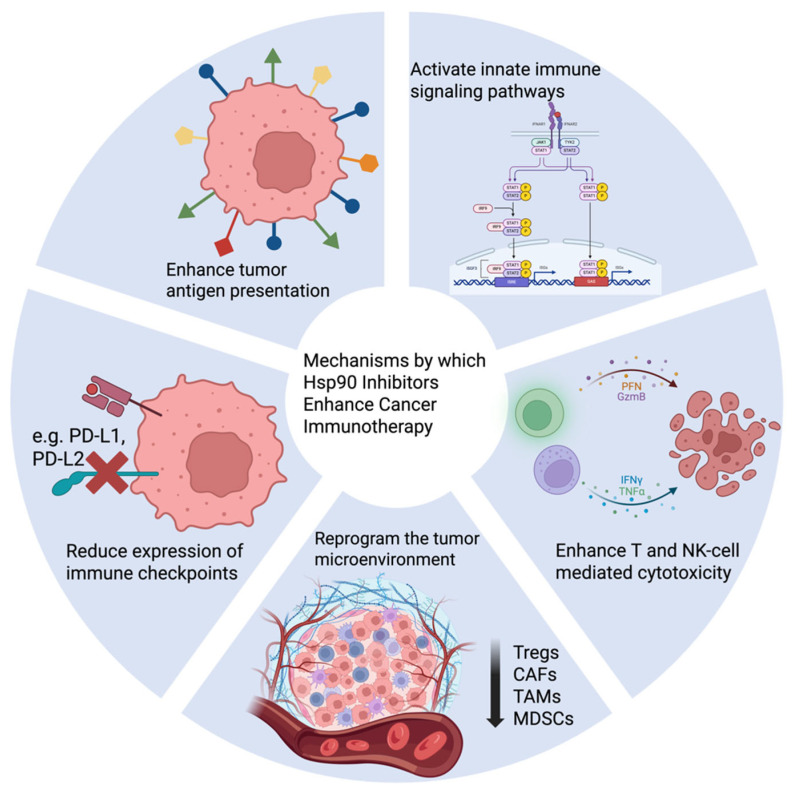
Mechanisms by which Hsp90 inhibitors enhance cancer immunotherapy. Hsp90 inhibition improves the efficacy of cancer immunotherapy through various mechanisms. These include enhanced tumor antigen presentation, decreased immune checkpoint expression, reprogramming of the tumor microenvironment, increased cytotoxicity mediated by T and NK cells, and activation of innate immune.

Recent literature has identified a number of non-mutually exclusive mechanisms underlying the immuno-modulatory activity of Hsp90 inhibitors and the enhanced immunotherapy efficacy in various preclinical models (Figure 17; Table 2).

*Enhanced tumor antigen presentation.* Tumor antigen presentation is critical for effective antitumor immunity, yet it is frequently compromised in cancer. One major immunomodulatory effect of Hsp90 inhibition involves improved tumor antigen processing and presentation. Pharmacologic blockade of Hsp90 transcriptionally upregulates melanocytic differentiation antigens such as gp100, Melan-A/MART-1, and TRP-2, expanding the intracellular antigen pool [137]. In parallel, Hsp90 inhibitors promote proteasome-dependent degradation of oncogenic client proteins such as EphA2, increasing antigenic peptides available for MHC-I loading [136,139]. Additionally, sustained low-dose Hsp90 inhibition has been found to amplify and diversify the MHC-I immunopeptidome through an IFNγ-independent mechanism involving upregulation of immunoproteasome components such as PSMB8, without inducing a heat shock response or impairing T cell function [138]. Collectively, these findings indicate that Hsp90 inhibition enhances tumor antigen presentation through multiple mechanisms, including transcriptional induction of differentiation antigens, degradation of client proteins, and proteasome-driven broadening of the antigenic repertoire.

*Reduction in immune checkpoint molecules.* Immune checkpoint molecules are primarily expressed on immune cells and play a crucial role in maintaining immune homeostasis. However, tumor cells can exploit these molecules to evade immune surveillance. For instance, PD-L1 on tumor cells binds to its receptor, PD-1, on T cells, transmitting inhibitory signals that suppress T cell cytotoxicity and thereby promote immune evasion. Hsp90 inhibition reduces the expression of critical immune checkpoint molecules on tumor cells, such as PD-L1 and PD-L2. This effect is primarily mediated by destabilizing key transcriptional regulators, including STAT3, STAT1, and c-Myc, which govern immune checkpoint gene expression [140,141,143,144]. In addition, PD-L1 expression is known to be regulated by several oncogenic Hsp90 client proteins, such as mutant EGFR, rearranged ALK, HIF-1α, and JAK2 [142]. By downregulating these immune checkpoint molecules, Hsp90 inhibition renders tumor cells more susceptible to immune attack. Accordingly, combining Hsp90 inhibitors with checkpoint blockade antibodies, particularly anti-PD-1, has demonstrated enhanced antitumor efficacy in preclinical models by alleviating tumor-mediated immune suppression and enhancing T cell cytotoxicity [143,144].

*Reprogramming of tumor microenvironment.* Beyond direct tumor cell effects, Hsp90 inhibitors target immunosuppressive cell populations (e.g., Tregs, MDSCs, TAMs, CAFs) and cytokines within the TME, shifting the balance toward antitumor immunity. In gastric cancer, pimitespib selectively degrades STAT5, thereby downregulating FOXP3 and inhibiting the proliferation and immunosuppressive function of FOXP3^high^ effector Tregs [145]. In pancreatic cancer, XL888 has been shown to suppress CAF-secreted IL-6, thus promoting T-cell infiltration and sensitizing tumors to PD-1 blockade [146]. Furthermore, Hsp90 inhibition prevents the tumor-induced differentiation of monocytes into immunosuppressive TAMs or MDSCs, thereby alleviating immune suppression in breast cancer and melanoma models [147,148].

*Enhancement of T and NK cell-mediated cytotoxicity*. Hsp90 inhibitors sensitize tumor cells to cytotoxic T lymphocyte (CTL) and natural killer (NK) cell-mediated killing through increased susceptibility and ligand expression. For instance, ganetespib enhances T cell-mediated cytotoxicity in melanoma by upregulating type I interferon-stimulated genes, particularly members of the IFIT family (*IFIT1*, *IFIT2*, *IFIT3*), thereby sensitizing tumor cells to immune attack. This effect improves efficacy when combined with anti-PD-1 and anti-CTLA-4 antibodies in preclinical models [149]. Similarly, BIIB021 upregulating NKG2D ligands, including MICA/B and ULBP2, on Hodgkin’s lymphoma cells, enhancing their susceptibility to NK cell-mediated cytotoxicity [150]. Moreover, combining Hsp90 inhibitors, such as 17-DMAG, with adoptive cellular immunotherapy, including cytokine-induced killer (CIK) cells, has been shown to significantly enhance therapeutic T cell responses and overcome tumor resistance [151,152]. One mechanism underlying this synergy involves the increased tumor cell susceptibility to Fas/FasL-mediated apoptosis induced by Hsp90 inhibition [152].

*Activation of innate immune pathways.* Recent evidence indicates that Hsp90 inhibition can activate innate immune signaling pathways, including the induction of ISGs and the expression of endogenous retroviral elements, thereby enhancing antitumor immunity through innate immune mechanisms [149,154]. Notably, Enniatin A, a natural Hsp90 inhibitor that does not induce a compensatory HSR, has been shown to trigger immunogenic cell death (ICD). ICD results in the release of damage-associated molecular patterns (DAMPs), leading to robust activation of innate immune responses. In preclinical models of triple-negative breast cancer, Enniatin A treatment was also associated with reduced PD-L1 expression and increased infiltration of cytotoxic CD8^+^ T cells [155].

*Combination with other therapeutic modalities.* In addition to sensitizing immunotherapy, Hsp90 inhibitors have demonstrated notable synergy when combined with other therapeutic modalities, including radiotherapy, targeted therapy, and photodynamic therapy. These combinations enhance systemic antitumor immunity, reduce resistance, and improve clinical responses [146,153,156,157,158]. For example, when 17-AAG was used in combination with the BRAF inhibitor, vemurafenib, it showed an inhibition of compensatory MAP kinase activation in melanoma cells, which resulted in enhancement of its towards cellular immunotherapy and checkpoint blockade [151]. Additionally, the combined inhibition of Hsp90 and caspase-9 has emerged as a compelling strategy to convert immunologically silent apoptosis into ICD. Hsp90 inhibition induces DNA fragmentation and apoptosis, while caspase-9 blockade activates intrinsic DNA sensing pathways via cGAS-STING, leading to interferon-β production and robust CD8^+^ T cell-mediated tumor control. This dual targeting approach triggers the release of damage-associated molecular patterns (DAMPs), enhances tumor immunogenicity, and synergizes with PD-L1 blockade to achieve complete tumor regression in preclinical models [159].

## 11. Grp94 Inhibition in Immune Regulation and Immunotherapy

Grp94 plays a multifaceted role in tumor immunity by chaperoning key immune-regulatory proteins, modulating antigen presentation, and shaping the TME. As an ER-resident HSP90 family member, Grp94 is essential for the proper folding and surface expression of integrins, Toll-like receptors, and GARP (the TGF-β-binding protein) on Tregs [160,161]. Through these clients, Grp94 supports tumor cell survival and the establishment of a local immunosuppressive niche. Grp94 also influences antigen presentation pathways. When tumor cells undergo stress or death, Grp94 bound to tumor-derived peptides can be released into the extracellular space. These Grp94-peptide complexes are recognized and internalized by antigen-presenting cells via receptors such as CD91, leading to cross-presentation on MHC I and activation of CD8^+^ T cells [162,163]. This natural adjuvant function of Grp94 has underpinned efforts to develop Grp94-based cancer vaccines [164].

Beyond its adjuvant role, however, the inhibition of Grp94 profoundly remodels the TME to favor immune activation. One hallmark of Grp94 blockade is a reversal of T-cell suppression within tumors. Grp94 activity in Tregs promotes their stability and immuno-suppressive function. Grp94 chaperones GARP, which anchors latent TGF-β on Treg surfaces, thereby facilitating TGF-β–mediated immunosuppression [161]. Grp94 also ensures high surface expression of the integrin LFA-1 (CD11a/CD18) on Tregs, which is crucial for their trafficking into tumors [165]. Consequently, inhibiting Grp94 can selectively debilitate Tregs: genetic ablation of Grp94 in Foxp3^+^ Tregs was shown to abolish Treg accumulation in the tumor microenvironment and to destabilize the Treg lineage, evidenced by reduced Foxp3 expression and conversion of some Tregs into interferon-γ–producing ex-Tregs [161,165]. One key mechanism is disruption of IL-2/STAT5 signaling in Tregs upon Grp94 loss; without Grp94, Tregs had markedly lower CD25 (IL-2Rα) expression and phosphorylated STAT5, impairing their activation [165].

Grp94 inhibition also targets immunosuppressive myeloid populations. In a 4T1 breast cancer model, treatment with a Grp94-selective inhibitor, PU-WS13, led to a marked reduction in CD206^+^ M2-polarized TAMs, accompanied by decreased intratumoral TGF-β levels and collagen deposition [166]. By disrupting Grp94-dependent folding of receptors and integrins required for TAM polarization and retention, this strategy interrupts the TGF-β-driven cycle of fibrosis, MDSC recruitment, and further Treg differentiation. The remodeled stroma becomes less fibrotic and more permissive to T cell infiltration, with treated tumors showing deeper penetration of CD8^+^ lymphocytes.

## 12. Hsp90α/β Inhibitors to Enhance Immunotherapy

Isoform-selective inhibitors targeting Hsp90α and/or Hsp90β have recently emerged as promising agents for enhancing the efficacy of cancer immunotherapy. Distinct from traditional *pan*-Hsp90 inhibitors, isoform-selective inhibitors can specifically disrupt individual isoforms of Hsp90, allowing targeted modulation of immune pathways with improved efficacy and reduced off-target toxicity.

The dual isoform-selective inhibitor of HSP90α/β, pimitespib (TAS-116), has demonstrated immunomodulatory activity through selective targeting of Tregs within the TME [145]. Tregs are potent mediators of immunosuppression and contribute to resistance against immune checkpoint blockade (ICB). Preclinical studies and analyses of clinical samples from gastric cancer patients have shown that pimitespib effectively reduces populations of highly immunosuppressive FOXP3^high^ effector Tregs (eTregs). Mechanistically, pimitespib promotes the degradation of STAT5, an essential mediator of IL-2 signaling critical for the survival, maintenance, and functional competence of Tregs. This selective depletion impairs Treg-driven immunosuppression without negatively impacting CD8^+^ effector T cell populations, thereby shifting the immune equilibrium toward antitumor immunity. Additionally, pimitespib enhances the priming and activation of antigen-specific CD8^+^ T cells, including responses directed against tumor-associated antigens such as NY-ESO-1. In murine tumor models, combining pimitespib with anti-PD-1 antibodies results in significantly enhanced tumor suppression and prolonged progression-free survival compared with monotherapy. Importantly, by targeting intratumoral Tregs, the method avoids the risk of systemic autoimmunity, a major drawback of traditional Treg-targeted therapies. Collectively, these findings highlight the potential of α/β isoform-selective Hsp90 inhibition, exemplified by pimitespib, as a promising strategy to overcome adaptive immunotherapy resistance through targeted modulation of immunosuppressive components in the TME.

In contrast to dual α/β inhibition, selective targeting of the Hsp90β isoform offers a mechanistically distinct approach to modulate tumor immunity. NDNB1182, a rationally designed Hsp90β-selective inhibitor, suppresses CDK4 expression without inducing a compensatory heat shock response—a limitation commonly observed with *pan*-Hsp90 inhibitors [154]. Mechanistic studies have shown that CDK4 downregulation leads to reduced DNMT1 expression, reactivated expression of endogenous retroviral elements (ERVs), and subsequent stimulation of interferon-stimulated genes. This cascade promotes a viral mimicry response that enhances type I interferon signaling and increases antigenicity within the TME. In mouse models of prostate and breast cancer, co-administration of NDNB1182 with PD-1 and CTLA-4 blockade led to improved tumor control compared to either treatment alone. This enhanced efficacy was associated with an increase in tumor-infiltrating CD8^+^ T cells and dendritic cells, suggesting that Hsp90β inhibition facilitates a more immunostimulatory microenvironment. Notably, NDNB1182 treatment did not induce observable systemic toxicity in vivo. This indicates that isoform-selective inhibition may mitigate the safety concerns of *pan*-Hsp90 inhibitors. These findings position Hsp90β-selective inhibition as a promising strategy to activate innate immunity and sensitize immunologically “cold” tumors to immune checkpoint therapies.

## 13. Clinical Trials Combining Hsp90 Inhibitors with Immunotherapy

Based on preclinical evidence supporting the immunomodulatory potential of Hsp90 inhibition, two early-phase clinical trials have been reported to combine Hsp90 inhibitors with anti-PD1 immunotherapy (Table 3).

In one phase Ib/II study, the combination of the pan-Hsp90 inhibitor XL888 and pembrolizumab was evaluated in patients with advanced colorectal cancer (CRC) [167]. While the combination was well tolerated, clinical responses were modest, with stable disease observed in a subset of patients with treatment-refractory, mismatch repair-proficient (pMMR) CRC. Notably, the correlative immune analysis showed that Hsp90 inhibition changed the tumor immune profile, with decreases in IL-6-producing cells and tumor-associated macrophages and system-wide increases in inflammatory cytokines, which implies enhanced immune activation.

A second trial evaluated TAS-116 (pimitespib), a selective Hsp90α/β inhibitor, in combination with nivolumab across multiple solid tumors, including microsatellite-stable (MSS) CRC [168]. This study observed an objective response rate (ORR) of 16% in MSS CRC patients without prior exposure to ICIs. This is an encouraging outcome given the known resistance of this population to PD-1 blockade. Mechanistically, TAS-116 was found to suppress Treg activity in both peripheral blood and tumor-infiltrating lymphocytes, reducing immunosuppressive pressure and potentially improving responsiveness to ICIs. The combination exhibited a manageable safety profile without unexpected toxicities. Pimitespib’s improved safety profile (as a second-generation Hsp90 inhibitor with less ocular and hepatic toxicity) likely contributed to the feasibility. This trial has prompted interest in further studies (possibly a randomized phase II/III) to see if Hsp90 inhibitors, in combination with PD-1 blockade, could benefit a subset of MSS CRC or other checkpoint-refractory solid tumors.

No clinical trials have explored Hsp90 inhibition in combination with CAR-T cells, oncolytic viruses, or cancer vaccines in solid tumors. However, these avenues are receiving growing interest. For example, Hsp90 inhibitors could be paired with anti-CD19 CAR-T therapy in aggressive lymphomas to improve T cell persistence or modulate the tumor microenvironment [169]. Similarly, Hsp90 inhibitors could potentially enhance oncolytic virus-based therapies by sustaining immune priming post-viral antigen release, although timing and toxicity remain important considerations.

Parallel efforts are also underway to develop isoform-selective Hsp90 inhibitors suitable for combination regimens. While pimitespib exhibits partial Hsp90α selectivity [168], novel compounds with high specificity for Hsp90β are in preclinical stages. This approach aims to degrade Hsp90β-dependent clients, such as CDK4 and IFN regulatory proteins, while minimizing off-target effects linked to Hsp90α inhibition in normal tissues [154]. NDNB1182 is one such Hsp90β-selective agent currently under investigation for its potential to stimulate innate immune signaling without eliciting a compensatory heat shock response. These pharmacological features may enable prolonged administration alongside immunotherapy with a wider therapeutic window.

Overall, initial clinical trials suggest the potential for combining Hsp90 inhibitors with immunotherapy regimens. Continued clinical investigation, particularly with isoform-selective agents, will be critical to determine whether Hsp90 inhibition can help expand the reach and durability of current immunotherapeutic strategies.

## 14. Summary and Future Directions

Isoform-selective Hsp90 inhibitors have emerged from systematic chemical modification and structural insights into the Hsp90 ATP-binding domain. Initially, the field focused on natural products such as geldanamycin and radicicol, which bind to the N-terminal ATP pocket but exhibit limited clinical utility due to hepatotoxicity and poor pharmacokinetics. Advances in medicinal chemistry led to derivatives like 17-AAG and synthetic benzamide-based scaffolds (e.g., SNX-2112, TAS-116), which improved drug-like properties but lacked isoform selectivity. Recent breakthroughs achieved selective targeting through careful structural analyses identifying subtle differences between isoforms, notably exploiting unique residues and sub-pocket conformations specific to Hsp90β and Grp94. Selective inhibitors such as KUNB31 and NDNB1182 were developed by modifying key functional groups to leverage these structural distinctions, avoiding toxicities linked to Hsp90α inhibition. Similarly, Grp94-selective inhibitors were designed by targeting unique insertion sequences and secondary binding pockets exclusive to this isoform, refining pharmacological specificity and therapeutic potential.

Pan-Hsp90 inhibitors broadly disrupt multiple signaling pathways but provoke compensatory heat shock responses and notable side effects, limiting their clinical utility. Nevertheless, they significantly enhance tumor immunity by increasing antigen presentation, reducing immune checkpoint expression, and remodeling immunosuppressive tumor microenvironments. Isoform-selective inhibitors like pimitespib (TAS-116) and NDNB1182 modulate tumor immunity more precisely. For example, pimitespib specifically impairs regulatory T-cell (Treg) functions through STAT5 degradation without triggering a systemic heat shock response, enhancing sensitivity to checkpoint inhibitors. NDNB1182 selectively targets Hsp90β, leading to reduced CDK4 levels, reactivation of endogenous retroviral elements, and subsequent innate immune stimulation, effectively converting immunologically “cold” tumors to “hot.” These inhibitors demonstrate a distinct mechanism that augments both innate and adaptive immunity without the detrimental effects observed with pan-inhibitors, presenting a promising approach to overcoming resistance mechanisms in cancer immunotherapy.

Currently, isoform-selective Hsp90 inhibitors show encouraging preclinical and initial clinical outcomes when combined with immunotherapies, particularly immune checkpoint blockade. Clinical studies combining pan-inhibitor XL888 or α/β-selective inhibitor TAS-116 with anti-PD-1 agents reported modest yet promising improvements in tumor immune infiltration and patient responses, particularly in immunotherapy-resistant tumors. The field now anticipates broader exploration of these inhibitors in combination with other immunotherapeutic strategies, including CAR-T cells, cancer vaccines, and oncolytic viruses.

Despite these advances, several challenges need to be addressed before Hsp90 isoform-selective inhibitors can be fully translated into clinical practice. Optimization of drug-like properties, such as solubility, permeability, metabolic stability, and isoform selectivity, will be essential to achieve selective inhibition while minimizing off-target toxicity. Our current understanding of how each isoform of Hsp90 regulates tumor progression and immune responses remains limited. Understanding isoform-specific roles will guide the design of more effective therapeutic strategies. Moreover, the mechanisms by which tumors may become resistant to Hsp90 isoform-selective inhibition remain poorly understood and require further study. This could involve a switch in reliance between isoforms or compensatory activation of alternative signaling pathways. The identification of clinically actionable biomarkers will also be crucial. Candidate biomarkers may include measurable changes in client protein levels, activation of endogenous retroviral elements, or distinctive immune-related signatures. Practical matters, including timing, dosing, and administration sequence, must be addressed through careful pharmacodynamic scheduling studies to optimize the combination of these inhibitors with checkpoint blockade or other immunotherapies.

Although the challenges persist, isoform-selective Hsp90 inhibition represents a promising strategy to fine-tune antitumor immunity with greater precision and reduced systemic toxicity. As we move toward next-generation immunotherapies, integrating these agents into personalized, biomarker-driven treatment regimens holds considerable promise for improving outcomes across malignancies.

## Figures and Tables

**Table 1:** Examples of Hsp90 client proteins associated with the hallmarks of cancer.

Hallmark of Cancer	Examples of Hsp90 Client Proteins
Sustaining proliferative signaling	c-Myc, STAT3, EGFR, HER2/ERBB2, Notch1, mTOR
Evading growth suppressors	p53, CDK4, CDK6, MDM2, MDM4
Avoiding immune destruction	STAT3, IDO1, IRAK3
Enabling replicative immortality	p23, c-Myc, Telomerase, FOXM1
Tumor-promoting inflammation	NF-κB, Hsp27, Hsp70, ASK1, IL-6, IL-8
Activating invasion and metastasis	c-Met, MMP2, MMP9, N-cadherin
Inducing or accessing vasculature	VEGFR, EGFR, HIF-1α, TBK1, FLT3
Genome instability and mutation	BRCA1, BRCA2, p53, ATM, Chk1, Chk2, NEK8, NEK9
Resisting cell death	p53, Survivin, BCL-2, BCL-XL, Akt, Hsp27, Hsp70
Deregulating cellular metabolism	c-Myc, PIK3CA, mTOR, Nrf2, HSF1, ARNT, ARRB1
Unlocking phenotypic plasticity	Nanog, Oct4, SOX11, EZH2
Epigenetic reprogramming	EZH2

The hallmarks of cancer, as conceptualized by Hanahan and Weinberg [17,19,20], define the essential biological capabilities acquired during tumor development. This table provides examples of Hsp90 client proteins that are linked to these hallmarks of cancer. A complete list of Hsp90-interacting proteins is available at https://www.picard.ch/downloads/Hsp90interactors.pdf (accessed on 16 June 2025).

**Table 2:** Mechanisms by which Hsp90 inhibition enhances immunotherapy.

Mechanism	Key Examples	Representative Hsp90 Inhibitors	Ref.
Enhanced antigen presentation	EphA2, gp100, Melan-A/MART-1 upregulation; Increased MHC-I expression	17-DMAG, Ganetespib	[136,137,138,139]
Immune checkpoint modulation	Reduced PD-L1, PD-L2 via STAT1/3, c-Myc inhibition	Ganetespib, XL888, Pimitespib	[140,141,142,143,144]
Tumor microenvironment reprogramming	Reduction in Tregs, MDSCs, TAMs, CAFs; Decreased immunosuppressive cytokines	XL888, Pimitespib, 17-DMAG	[145,146,147,148]
Enhanced T and NK cell-mediated cytotoxicity	Improved T/NK cell activity via increased tumor susceptibility	Ganetespib, BIIB021, 17-DMAG	[136,142,149,150,151,152,153]
Activation of innate immune signaling pathways	Induction of ICD, cGAS-STING activation, interferon signaling pathways activation, induce the expression of endogenous retroviral elements	Ganetespib, NDNB1182, Enniatin A	[149,154,155]

**Table 3:** Clinical trials combining Hsp90 inhibitors with immunotherapy.

Trial ID	Hsp90 Inhibitor	Immunotherapy	Cancer Type	Phase	Key Findings	Clinical Outcome	Ref.
NCT03095781	XL888 (pan-Hsp90)	Pembrolizumab	pMMR Colorectal Cancer	Ib/II	Modulated TME by reducing IL-6^+^ cells and macrophages; increased systemic cytokines	Stable disease in subset; no ORR	[167]
EPOC1704	TAS-116 (Hsp90α/β selective)	Nivolumab	MSS Colorectal Cancer	Ib	Reduced Treg activity in PBMCs and TILs; enhanced immune activation	ORR 16% in MSS CRC	[168]

Abbreviations: pMMR, proficient mismatch repair; TME, tumor microenvironment; ORR, objective response rate; MSS, microsatellite stable; PBMCs, peripheral blood mononuclear cells; TILs, tumor-infiltrating lymphocytes.
